# Mackintosh’s Practice of Medicine

**Published:** 1844-09

**Authors:** 


*Mackintosh's Practice of Medicine.*—Lindsay & Blakiston of Philadelphia, are about publishing a fourth edition of this valuable work, much used as a text book in the medical schools of the United States. It is to be brought up to the present time, with notes and additions by Samuel George Morton, M. D., a gentleman well known for his high professional attainments, and late physician to the Philadelphia Hospital, &c. We have no doubt that the work will meet with a good reception from the profession and medical students.—[Ed.

